# TRP-Dependent Calcium Regulation in HCEC-12 Cells: Involvement of Ascorbic Acid and Cannabinoid Receptor Signaling

**DOI:** 10.3390/ijms27093902

**Published:** 2026-04-28

**Authors:** Louay Homsi, Anisha Atul Bhamare, Uwe Pleyer, Stefan Mergler

**Affiliations:** 1 Department of Ophthalmology, Charité–Universitätsmedizin Berlin, Corporate Member of Freie Universität Berlin and Humboldt-Universität zu Berlin, 13353 Berlin, Germany; louay.homsi@charite.de (L.H.); anisha-atul.bhamare@charite.de (A.A.B.); uwe.pleyer@charite.de (U.P.); 2 Berlin Institute of Health, Charité–Universitätsmedizin Berlin, 10178 Berlin, Germany

**Keywords:** human corneal endothelial cells (HCEC-12), L-ascorbic acid (Asc), TRP channels, cannabinoid receptor 1 (CB1), intracellular calcium homeostasis, calcium imaging, patch-clamp, WIN 55,212-2, human corneal endothelium

## Abstract

The human corneal endothelium (HCE) is critical for maintaining corneal transparency. Dysfunctions due to cell loss are linked to altered intracellular calcium ([Ca^2+^]_i_) homeostasis. Transient receptor potential channels (TRPs) are key regulators of [Ca^2+^]_i_, and both L-ascorbic acid (Asc) and cannabinoid receptor (CB) agonists have been implicated in modulating TRP activity. This study investigated the effects of 1 mM Asc and the CB agonist WIN 55,212-2 (WIN) (10 µM) on [Ca^2+^]_i_ regulation in human corneal endothelial cells (HCECs). HCEC-12 was used as the established HCE cell model. [Ca^2+^]_i_ dynamics were assessed by fura-2/AM fluorescence imaging, and membrane currents were analyzed using planar patch-clamp recordings. Adding 1 mM Asc increased [Ca^2+^]_i_, which was partially suppressed by the TRPV1 blocker AMG-9810 (AMG) (20 µM) and the TRPV4 blocker GSK2193874 (GSK219) (10 µM). Furthermore, 1 mM Asc increased whole-cell currents. WIN also induced [Ca^2+^]_i_ transients that were partially attenuated by AMG, the TRPM8 blocker AMTB (20 µM), GSK219, and the CB1 inverse agonist AM251 (10 µM). In addition, combined treatment with Asc and WIN enhanced [Ca^2+^]_i_ elevations compared with either treatment alone. These findings provide the first evidence for a functional interaction between TRP channel activity and CB signaling in HCECs. The inhibitory effect of AM251 suggests a predominant contribution of CB1 receptors. Given the central role of Ca^2+^ homeostasis in corneal endothelial function and disease, these results may contribute to a better understanding of endothelial pathophysiology and support further investigation of TRPs and cannabinoid signaling as potential targets in corneal disorders.

## 1. Introduction

The cornea consists of several layers, with the corneal epithelium being the outermost cell layer, followed by Bowman’s membrane, the corneal stroma, the Descemet membrane and the corneal endothelium, which is in direct contact with the aqueous humor in the anterior chamber of the eye [1]. It is composed of a monolayer of cells forming a hexagonal pattern [2]. These cells lack mitotic activity in humans, meaning that damaged cells cannot be physiologically replaced [3,4,5]. The corneal endothelium plays a crucial role in maintaining corneal transparency, as first demonstrated by Maurice [6]. It regulates water homeostasis between the corneal stroma and the aqueous humor, ensuring adequate nutrition for the cornea [7,8]. Disruption of this homeostasis leads to the accumulation of water and solutes in the stroma, resulting in corneal swelling and a subsequent loss of transparency [9]. Human corneal endothelial cell density exhibits an age-related decline [10]. Certain conditions can lead to an exacerbation of this cell loss including diseases like Fuchs endothelial dystrophy [11]. Evidence suggests that L-ascorbic acid (Asc) can ameliorate dysfunction in the rabbit corneal endothelium and enhance proliferation in immortalized human corneal endothelial cells (B4G12 cells) [12].

L-ascorbic acid performs multiple physiological functions in the human body, with well-known roles in collagen synthesis and as a component of the antioxidative system [13,14]. Evidence suggests that Asc promotes wound healing in the human corneal epithelium and is associated with improved outcomes in alkali burns of the cornea [15]. Notably, Asc concentrations in mammalian tissues—particularly in the bovine cornea—are substantially higher than those found in serum. Moreover, the bovine corneal epithelium exhibits especially high Asc levels, ranging from 1.39 to 1.56 mg/g. In contrast, the bovine stroma, endothelium, and aqueous humor have a lower concentration of approximately 0.2 mg/mL [16]. In multiple previous studies 1 mM Asc was used in human corneal endothelial cells (HCECs) to examine effects on cell survival and proliferation [12,17]. Various experiments suggest that the relatively high concentration of Asc in mammalian corneal cells and the aqueous humor is mediated through transporters such as Glucose transporter 1 (GLUT1) or Sodium-dependent Vitamin C Transporter 2/1 [18,19,20,21]. In retinoblastoma cells, Asc has been shown to affect Ca^2+^ homeostasis involving transient receptor potential channels (TRPs) [22].

Calcium signaling plays a central role in the regulation of proliferation, apoptosis, metabolism, and many other cellular functions [23]. Intracellular Ca^2+^ ([Ca^2+^]_i_) homeostasis is determined by the balance between Ca^2+^ influx, Ca^2+^ efflux, and release from intracellular stores [23]. These processes are regulated by multiple classes of ion channels and transport systems, including TRPs, voltage-gated Ca^2+^ channels, and Ca^2+^ pumps [24,25,26,27].

The TRPs superfamily comprises seven major subfamilies: TRPA (ankyrin), TRPC (canonical), TRPM (melastatin), TRPML (mucolipin), TRPN (no mechanoreceptor potential C), TRPP (polycystin), and TRPV (vanilloid). These channels are permeable to mono- and divalent cations, including Ca^2+^ [28]. They can be activated by various stimuli, including heat, cold, mechanical forces, different pH values, osmotic changes, and specific ligands, and can be triggered by G-protein-coupled receptors (GPCRs) (GPCR-TRPs axis) [24,29]. In excitable cells, such as neurons and cardiac cells, TRPs play a role in depolarization in connection with triggering action potential. In non-excitable cells, like fibroblasts or endothelial cells, they can influence cell signaling pathways [30]. The activation of TRPV1 channels elicits [Ca^2+^]_i_ transients that mediate mitogen-activated protein kinase (MAPK) pathway activation, thereby inducing IL-6 and IL-8 release in mammalian corneal epithelial cells [31]. Mergler et al. demonstrated the functional expression of various TRPs in HCECs, including TRPV1, TRPV4, and TRPM8 [32,33,34]. In HCECs it was shown that various stimuli, including heat and specific agonists, activate TRPV channels 1–3, supporting the hypothesis that these channels play essential homeostatic roles in HCECs, particularly in adapting to changing environmental conditions [32]. In particular, the TRPV4 channel appears to play a physiological role in osmosensing by responding to changes in cell volume and contributing to the maintenance of corneal transparency. In chondrocytes isolated from male Sprague–Dawley rats (rat model), it has been shown that the TRPV5 channel can activate Ca^2+^/calmodulin-dependent protein kinase II (CaMK2) through an increase in [Ca^2+^]_i_, leading to the activation of the MAPK pathway and the phosphorylation of extracellular signal-regulated kinases 1/2 (ERK1/2) [35].

Cannabinoid receptor 1 (CB1) is expressed in several ocular tissues and may contribute to corneal wound healing and the regulation of inflammation [36,37]. In addition, cannabinoid receptor (CB) signaling has been implicated in the regulation of Ca^2+^ homeostasis and in functional interactions with TRPs in corneal cells [38]. Cannabinoid receptors belong to the family of GPCRs, with CB1 and cannabinoid receptor 2 (CB2) being the most well-known members. These receptors are expressed in various tissues, including both neuronal and non-neuronal tissues [39]. Corneal tissues such as the corneal endothelium and epithelium, express CB1 [40]. Cannabinoid receptors are involved in the modulation and protection of various ocular tissues [41,42]. These receptors are physiologically activated by endocannabinoids and play a significant role in anti-inflammatory responses [37,43]. Several studies have demonstrated interactions between CB and TRPs, such as TRPA1, TRPV1, TRPV2, TRPV3, and TRPV4. The interaction between CB and TRPV1 is the most thoroughly described [38,39,44]. Evidence suggests that CB activation leads to TRPV1 phosphorylation through protein–protein interactions, resulting in channel desensitization. This ultimately reduces Ca^2+^ influx and the inflammatory response [37,44]. Interestingly, interactions with MAPK signaling, including ERK1/2 activation, have been reported in corneal epithelial cells following CB1 and TRPV1 activation, where these pathways are associated with downstream inflammatory responses and EGFR transactivation [37,45,46]. Some in vivo studies suggest the potential of CB agonists, such as WIN 55,212-2 (WIN), in the treatment of corneal injuries [36]. However, the effects of CB signaling on corneal endothelial cells under physiological and pathological conditions remain poorly understood. Given the limited therapeutic options for corneal endothelial dysfunction, understanding TRP- and CB-mediated Ca^2+^ signaling may provide a basis for the development of novel pharmacological strategies aimed at preserving endothelial function.

This study investigated the effects of Asc and the CB agonist WIN on Ca^2+^ homeostasis in cultivated HCEC-12 cells. Specifically, we aim to elucidate the mechanisms underlying Asc- and WIN-induced calcium signaling, focusing on the involvement of TRPV1, TRPM8, and TRPV4 channels. In addition, we address whether Asc-induced Ca^2+^ responses differ depending on exposure conditions, with particular consideration of acute versus prolonged treatment and subsequent washout.

## 2. Results

### 2.1. TRPV1 and TRPV4 Channel Blockers Partially Suppressed the Asc-Induced [Ca^2+^]_i_ Increase

**Figure 1:**
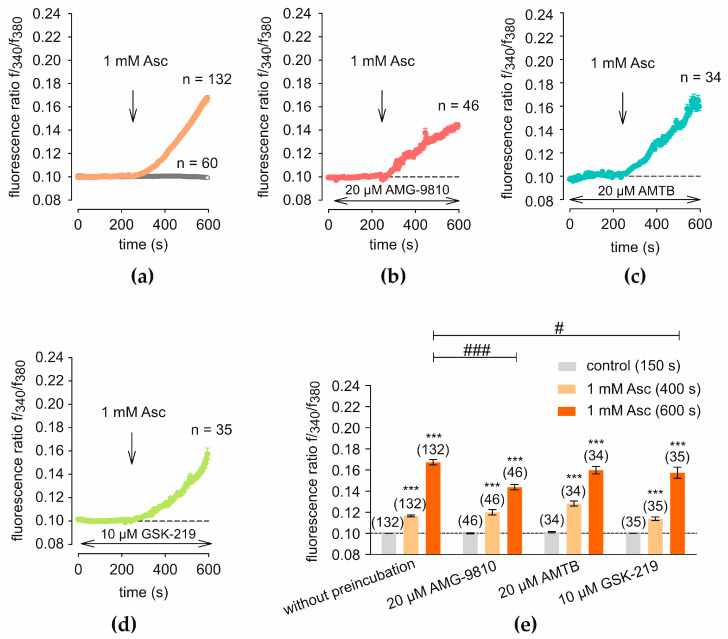
L-ascorbic acid (Asc)-induced [Ca^2+^]_i_ increase is modulated by transient receptor potential channels (TRPs) blockers. The mean fura-2/AM f340/f380 ratio (f340/f380) (ordinate) is plotted against time in seconds (abscissa), with an increase in f340/f380 correlating to an elevation in intracellular calcium ([Ca^2+^]_i_) concentration. The fluorescence ratios were normalized to baseline conditions (control set to 0.1, dashed line). At 240 s, 1 mM Asc is added (arrow). (**a**) Treatment with 1 mM Asc (*n* = 132) (filled circles) results in a steady increase in f340/f380 compared to the control condition without Asc treatment (*n* = 60) (open circles). (**b**) Preincubation with 20 µM AMG-9810 (AMG) (*n* = 46) leads to a reduction of absolute [Ca^2+^]_i_ following Asc treatment. (**c**) Preincubation with 20 µM AMTB (*n* = 34), a specific TRPM8 blocker, results in no significant changes of [Ca^2+^]_i_ compared to measurements without blocker. (**d**) Preincubation with 10 µM GSK2193874 (GSK219) (*n* = 35) results in a lower maximum of [Ca^2+^]_i_ compared to (**a**). (**e**) The diagram shows the mean f340/f380 at 150 s, 400 s, and 600 s and standard error (SEM) for each measurement in Figure 1. The numbers in brackets above the columns indicate the number of single cells measured (n), with each cell defined as an individual region of interest (ROI) selected during fluorescence microscopy. Asterisks (*) indicate statistical significance in paired data (*** *p* < 0.001), whereas hashtags (#) indicate statistical significance in unpaired data (# *p* < 0.05, ### *p* < 0.001). Statistical significance was defined as *p* < 0.05.

In the test solution, 1 mM Asc was used. Measurements were recorded continuously at 5 s intervals over a total duration of 600 s, and the time points at 150 s, 400 s, and 600 s were selected as representative values for baseline, post-treatment response, and the end of the recording period for statistical analysis. Asc was applied at *t* = 240 s to ensure a stable baseline prior to stimulation. This treatment resulted in an immediate and pronounced increase in the Fura-2/AM f340/f380 fluorescence ratio (f340/f380). The mean f340/f380 increases from 0.1000 ± 0.0001 (*t* = 150 s (control); *n* = 132) to 0.1165 ± 0.0009 (*t* = 400 s after the addition of Asc; *n* = 132; *p* < 0.001; Figure 1a), reaching a maximum of 0.1673 ± 0.0025 (*t* = 600 s; *n* = 132; *p* < 0.001; Figure 1a). Blockade of TRPV1 and TRPV4 channels leads to a partial reduction of Asc-induced increases in [Ca^2+^]_i_, as evidenced by experiments using channel-specific blockers. The concentrations of the TRP channel blockers used in this study (AMG, AMTB, and GSK-219) were selected based on previously established concentrations commonly applied in Ca^2+^ imaging experiments to achieve effective channel inhibition [47,48]. Preincubation with 20 µM AMG-9810 (AMG) results in a reduced slope of the curve, reaching a lower f340/f380 maximum of 0.1437 ± 0.0026 (*t* = 600 s; *n* = 46; Figure 1b), compared to the measurement without a blocker, which reaches 0.1673 ± 0.0025 (*t* = 600 s; *n* = 132; *p* < 0.001; Figure 1a). In contrast, measurements following preincubation with 20 µM AMTB, a specific TRPM8 blocker, show no delayed increase in f340/f380. The observed value of 0.1282 ± 0.0024 (*t* = 400 s; *n* = 34; Figure 1c) is not significantly lower than the value obtained with 1 mM Asc alone, which is 0.1165 ± 0.0009 (*t* = 400 s; *n* = 132; *p* < 0.001; Figure 1a). Preincubation with 10 µM GSK2193874 (GSK219), a TRPV4-specific blocker, similarly causes a delay in f340/f380 increase and leads to a lower maximum of 0.1574 ± 0.0052 (*t* = 600 s; *n* = 35; Figure 1d) compared to the control measurement, which reaches 0.1673 ± 0.0025 (*t* = 600 s; *n* = 132; *p* < 0.001; Figure 1a).

### 2.2. Washout Following 24-Hour Preincubation of 1 mM Asc in HCEC-12 Induces [Ca^2+^]_i_ Increases

**Figure 2:**
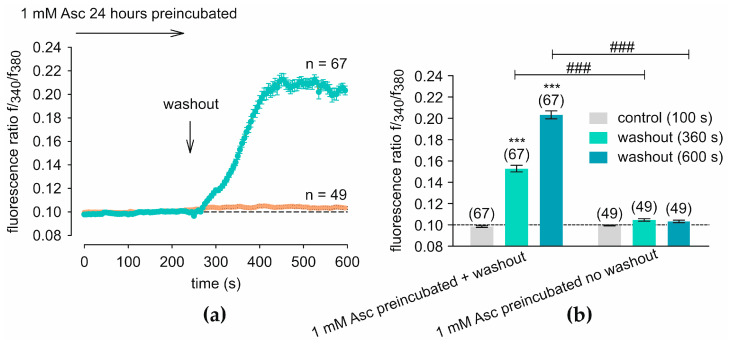
Washout of 24 h preincubated Asc induced [Ca^2+^]_i_ increase. The fluorescence ratios were normalized to baseline conditions (control set to 0.1, dashed line). (**a**) From 240 s (arrow), Asc is washed out with Ringer-Like-Solution (RLS) (Asc-free) (*n* = 67, green filled circles), leading to a notable increase in f340/f380 compared to the control measurement without washout. In this control measurement, the 1 mM Asc solution remained present and was not washed out (*n* = 49, orange filled circles). (**b**) A statistical analysis of the measurements shown in (**a**) is presented. Mean values ± SEM are shown at 100 s, 360 s, and 600 s. The numbers in brackets above the columns indicate the number of single cells measured (*n*). Asterisks (*) indicate statistical significance in paired data (*** *p* < 0.001), whereas hashtags (#) indicate statistical significance in unpaired data (### *p* < 0.001). Statistical significance was defined as *p* < 0.05.

HCEC-12 cells were pre-incubated with 1 mM Asc for 24 h (Figure 2a). The Asc-containing test solution was then washed out with the control solution (RLS) at 240 s, leading to a considerable increase in the f340/f380 from 0.0984 ± 0.0008 (*t* = 100 s before washout; *n* = 67) to 0.1528 ± 0.0031 (*t* = 360 s after washout; *n* = 67; *p* < 0.001), reaching a plateau at 0.2003 ± 0.0040 (*t* = 600 s; *n* = 67; *p* < 0.001). In the control measurement, where the 1 mM Asc solution was not washed out, no significant increase in [Ca^2+^]_i_ was observed (Figure 2a).

### 2.3. Asc-Induced [Ca^2+^]_i_ Increase Is Suppressed in Ca^2+^-Free Solution

**Figure 3:**
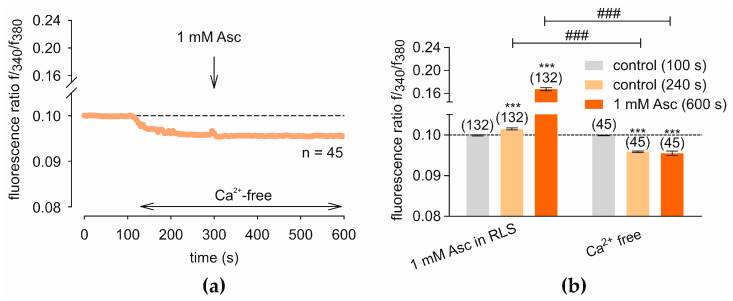
Asc-induced Ca^2+^ responses are absent in Ca^2+^-free solution. The fluorescence ratios were normalized to baseline conditions (control set to 0.1, dashed line). (**a**) At 120 s, the control solution was replaced with a Ca^2+^-free solution (5 mM EGTA), (*n* = 45), resulting in a slight decrease in the baseline level. At 300 s, 1 mM Asc was added to the Ca^2+^-free solution (arrow), which did not induce an increase in [Ca^2+^]_i_. (**b**) A statistical analysis of the measurements shown in panel (**a**) is presented. The response to 1 mM Asc in RLS (Figure 1a) is compared with that in Ca^2+^-free solution (**a**). Mean ± SEM are shown at 100 s, 240 s, and 600 s. The numbers in brackets above the columns indicate the number of single cells measured (n). Asterisks (*) indicate statistical significance in paired data (*** *p* < 0.001), whereas hashtags (#) indicate statistical significance in unpaired data (### *p* < 0.001). Statistical significance was defined as *p* < 0.05.

The Asc-mediated increase in [Ca^2+^]_i_ is dependent on extracellular calcium, as indicated by the measurements in Figure 3a. In the presence of a Ca^2+^-free solution containing 5 mM EGTA, applied at t = 120 s, the baseline f340/f380 decreases slightly from 0.0999 ± 0.001 (*t* = 100 s; *n* = 45) to 0.0960 ± 0.0002 (*t* = 240 s; *n* = 45; *p* < 0.001). After stabilization of the baseline under Ca^2+^-free conditions, 1 mM Asc was added at *t* = 300 s. This did not result in a significant change, with f340/f380 being 0.0955 ± 0.0005 (*t* = 600 s; *n* = 45; *p* > 0.05).

### 2.4. Asc Increases Whole-Cell Currents in HCEC-12

**Figure 4:**
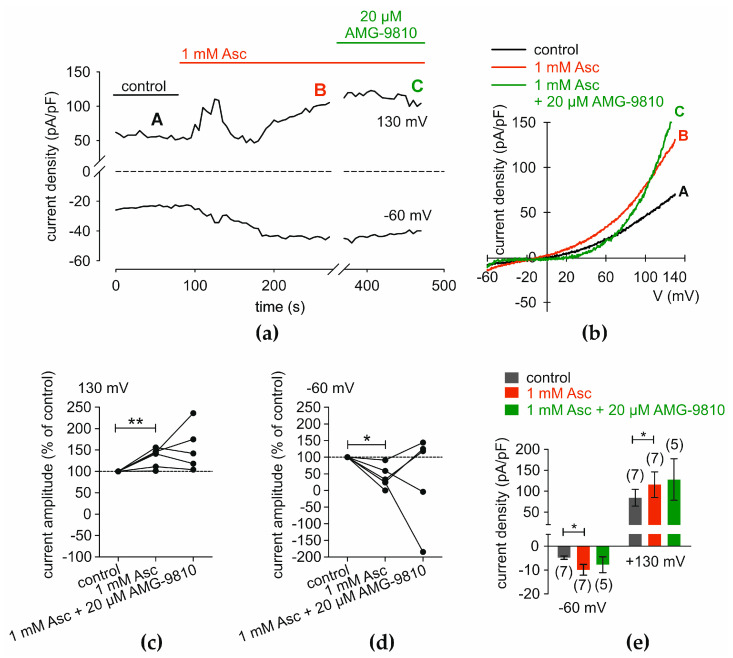
Asc-induced increases in whole-cell currents partially inhibited by AMG. Patch-clamp experiments demonstrate an increase in whole-cell currents following treatment with 1 mM Asc. (**a**) The time course of maximal outward (at 130 mV) and inward (at −60 mV) current densities, normalized to cell membrane capacitance, is plotted against time (in seconds). The dashed line indicates a change in axis. The first 100 s represent control currents (black bar). The red bar represents the presence of 1 mM Asc, and the green bar represents the presence of 20 µM AMG. (**b**) Current densities are plotted against membrane voltage. The letters indicate the time points in (**a**) at which the current–voltage analyses were performed. (**c**) A diagram showing single outward current amplitudes at 130 mV, expressed as a percentage of control (set to 100%), for measurements with 1 mM Asc and 1 mM Asc + 20 µM AMG. (**d**) A diagram equivalent to (**c**) regarding inward currents at –60 mV. (**e**) Statistical analysis of mean current amplitudes shown as current densities (pA/pF) at 130 mV and −60 mV. The number in brackets indicates the number of measurements. Asterisks (*) indicate statistical significance in paired data (* *p* < 0.05, ** *p* < 0.01). Statistical significance was defined as *p* < 0.05.

Patch-clamp measurements demonstrate an Asc-induced increase in whole-cell currents, as shown in Figure 4, suggesting the potential involvement of the TRPV1 channels in these currents. The time course of single-cell measurements reveals an increase in inwards and outwards current densities upon 1 mM Asc treatment. This increase is altered and partially reversed by the subsequent treatment with 20 µM AMG (Figure 4a). Whole-cell current increases are also evident in the current density–voltage relationship diagram, where current densities are higher in the presence of Asc compared to control measurements. The effect of 20 µM AMG on Asc-induced currents, however, is not entirely consistent. Inward currents and outward currents up to + 60 mV are completely reversed by 20 µM AMG. In contrast, no significant effect of AMG is observed for voltages exceeding + 60 mV (Figure 4b). Single outward current amplitudes showed in presence of 1 mM Asc a tendency toward higher values, whereas measurements with Asc and AMG display greater variability (Figure 4c). Single inward current amplitudes are generally higher in presence of Asc compared to control measurements, while addition of AMG yields overall lower inward currents than those observed with Asc alone. Two outliers are seen in this dataset indicating possible increase of leak inward currents during the measurements (Figure 4d). Outward current densities increased following the addition of 1 mM Asc from 84.50 ± 19.94 pA/pF (*n* = 7) to 115.77 ± 30.42 pA/pF (*n* = 7; *p* < 0.05; Figure 4e). Asc also induced an elevation in inward current densities, with values rising from −4.83 ± 1.85 pA/pF (*n* = 7) to −9.90 ± 5.84 pA/pF (*n* = 7; *p* < 0.05; Figure 4e).

### 2.5. CB1 Agonist WIN Induces [Ca^2+^]_i_ Transients, Which Were Partially Blocked by TRPV1, TRPM8, TRPV4 Blockers and CB1 Antagonist

**Figure 5:**
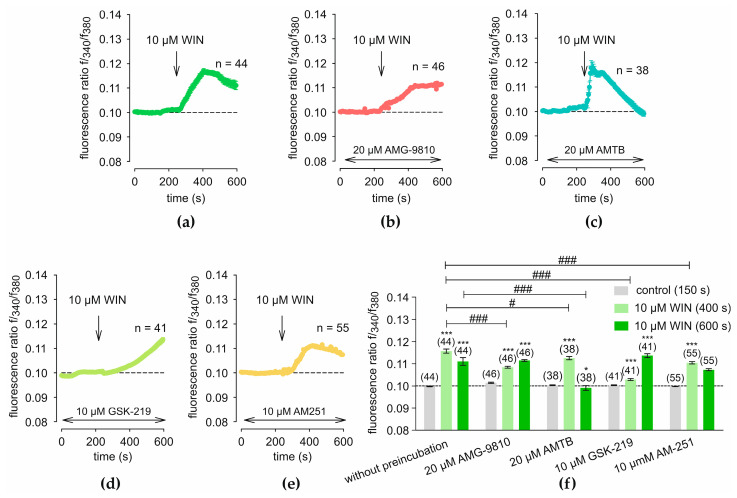
WIN 55,212-2 (WIN)-induced Ca^2+^ responses and modulation by TRP channel blockers and AM251. The point in time at which WIN was added is shown by an arrow. The fluorescence ratios were normalized to baseline conditions (control set to 0.1, dashed line). (**a**) Treatment with WIN at 240 s leads to a transient increase in f340/f380 (*n* = 44). (**b**) Preincubation with 20 µM AMG results in a reduced increase in f340/f380 (*n* = 46). (**c**) Preincubation with 20 µM AMTB results in a stronger recovery compared to that in (**a**) (*n* = 38). (**d**) Preincubation with 10 µM GSK219 causes a delay in the increase of the Fura f340/f380 compared to (**a**) (*n* = 41). (**e**) Preincubation with 10 µM AM251 results in a lower maximum f340/f380 compared to the control measurement (*n* = 55). (**f**) A statistical analysis of the measurements shown in panels (**a**–**e**) is presented. Mean values ± SEM are shown at 150 s, 400 s, and 600 s. The numbers in brackets above the columns indicate the number of single cells measured (*n*). Asterisks (*) indicate statistical significance in paired data (* *p* < 0.05, *** *p* < 0.001), whereas hashtags (#) indicate statistical significance in unpaired data (# *p* < 0.05, ### *p* < 0.001). Statistical significance was defined as *p* < 0.05.

Treatment with 10 µM WIN, a concentration commonly used to achieve effective activation of CB in Ca^2+^ imaging experiments [49], led to a [Ca^2+^]_i_ transient in HCEC-12 cells (Figure 5a). After baseline measurement (240 s), treatment with 10 µM WIN results in an initial rise in f340/f380 from 0.0998 ± 0.0002 (*t* = 150 s; *n* = 44; Figure 5a) to 0.1157 ± 0.0010 (*t* = 400 s following WIN addition; *n* = 44; *p* < 0.001; Figure 5a), followed by partial recovery, reaching a f340/f380 value of 0.1110 ± 0.0017 (*t* = 600 s; *n* = 44; *p* < 0.001; Figure 5a). These increases were partially reduced by specific TRP channel blockers. Specifically, preincubation with 20 µM AMG delays the f340/f380 increase, achieving a value of 0.1084 ± 0.0004 (*t* = 400 s; *n* = 46; Figure 5b) compared to 0.1157 ± 0.0010 (*t* = 400 s; *n* = 44; *p* < 0.001; Figure 5a) in the control measurement. Additionally, the f340/f380 does not recover but instead stabilizes at a plateau around 0.1114 ± 0.0004 (*t* = 600 s; *n* = 46; Figure 5b). Preincubation with 20 µM AMTB results in a calcium transient with a stronger recovery even below the baseline compared to the control measurement without blocker. Furthermore, with AMTB, recovery occurs more rapidly, returning to baseline values around 0.0991 ± 0.0011 (*t* = 600 s; *n* = 38; Figure 5c) compared to the control measurement value of 0.1110 ± 0.0017 (*t* = 600 s; *n* = 44; *p* < 0.001; Figure 5a). Comparable to the observations in Figure 5b, preincubation with 10 µM GSK219 delays the f340/f380 increase, with a value of 0.1029 ± 0.0004 (*t* = 400 s; *n* = 41; Figure 5d) compared to 0.1157 ± 0.0010 (*t* = 400 s; *n* = 44; *p* < 0.001; Figure 5a) in the control measurement. The f340/f380 demonstrates a steady increase without recovery, reaching 0.1136 ± 0.0002 (*t* = 600 s; *n* = 41; Figure 5d) compared to the baseline f340/f380 of 0.1003 ± 0.0002 (*t* = 150 s before WIN addition; *n* = 41; *p* < 0.001; Figure 5d). Preincubation with 10 µM AM251, a specific CB1 inverse agonist, which functionally inhibits CB1-mediated signaling, results in a delayed f340/f380 increase while maintaining the overall pattern observed in the control measurement. Consequently, the value of 0.1104 ± 0.0005 (*t* = 400 s; *n* = 55; Figure 5e) is lower than the corresponding control measurement value of 0.1157 ± 0.0010 (*t* = 400 s; *n* = 44; *p* < 0.001; Figure 5a). A statistical analysis of the measurements is presented in Figure 5f, showing mean values at 150 s, 400 s, and 600 s and confirming the significant differences between control conditions and the respective blocker treatments.

### 2.6. Asc and WIN Induce Significant Increases in [Ca^2+^]_i_ Partially Suppressed by TRPM8, TRPV4, and CB1 Antagonists

**Figure 6:**
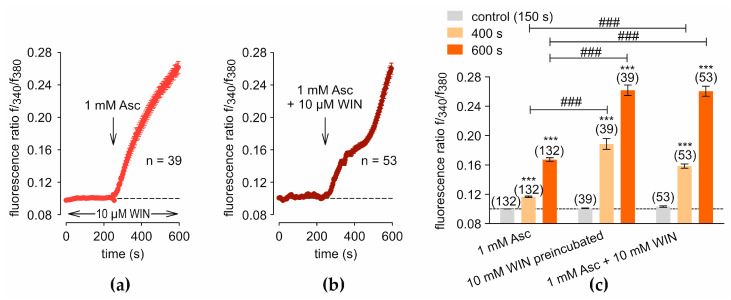
Combined effects of Asc and WIN on intracellular Ca^2+^ dynamics. The fluorescence ratios were normalized to baseline conditions (control set to 0.1, dashed line). (**a**) Preincubation with 10 µM WIN followed by treatment with 1 mM Asc at 240 s (*n* = 39) (arrow) leads to a steady increase in f340/f380 with decreasing slope. (**b**) Co-treatment with WIN and Asc at 240 s (*n* = 53) results in a biphasic and steep increase in [Ca^2+^]_i_. (**c**) The diagram presents a statistical analysis of the experiments shown in panels (**a**,**b**), compared with the response to 1 mM Asc alone (Figure 1a). Mean values ± SEM are shown at 150 s, 400 s, and 600 s. The numbers in brackets above the columns indicate the number of single cells measured (*n*). Asterisks (*) indicate statistical significance in paired data (*** *p* < 0.001), whereas hashtags (#) indicate statistical significance in unpaired data (### *p* < 0.001). Statistical significance was defined as *p* < 0.05.

Asc and WIN, when added together, induce substantial additive increases in [Ca^2+^]_i_, surpassing the individual effects of each compound (Figure 6). Preincubating with 10 µM WIN, rather than co-applying it with 1 mM Asc, produces a distinct pattern. In this scenario, f340/f380 increases steeply, rising from 0.1009 ± 0.0006 (*t* = 150 s; *n* = 39; Figure 6a) to 0.1884 ± 0.0074 (*t* = 400 s after 1 mM Asc addition; *n* = 39; *p* < 0.001; Figure 6a) and reaching 0.2616 ± 0.0072 (*t* = 600 s; *n* = 39; *p* < 0.001; Figure 6a), compared to measurements with Asc alone, which yield f340/f380 of 0.1165 ± 0.0009 (*t* = 400 s; *n* = 132; *p* < 0.001; Figure 1a) and 0.1673 ± 0.0025 (*t* = 600 s; *n* = 132; *p* < 0.001; Figure 1a). Additionally, f340/f380 values appear to approach a plateau. The simultaneous treatment with 10 µM WIN and 1 mM Asc results in a biphasic increase in f340/f380, with an exponential increase observed in the second phase. The f340/f380 rises from 0.1033 ± 0.0010 (*t* = 150 s; *n* = 53; Figure 6b) to 0.1583 ± 0.0030 (*t* = 400 s after drug treatment; *n* = 53; *p* < 0.001; Figure 6b) and further to 0.2603 ± 0.0068 (*t* = 600 s; *n* = 53; *p* < 0.001; Figure 6b). These values exceed those measured with 1 mM Asc alone, where the f340/f380 reaches 0.1673 ± 0.0025 (*t* = 600 s; *n* = 132; *p* < 0.001; Figure 1a).

**Figure 7:**
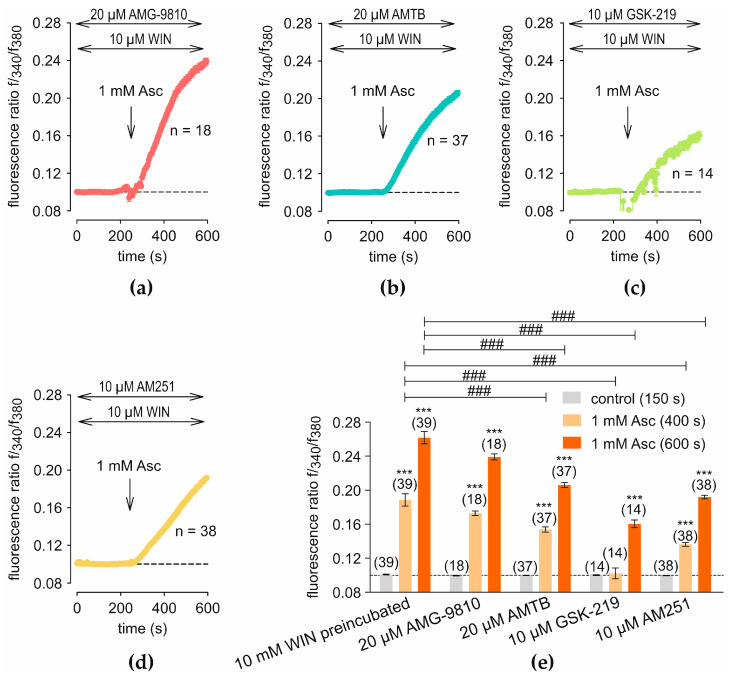
Inhibition of combined WIN- and Asc-induced Ca^2+^ responses by TRP channel blockers and AM251. The experimental protocol shown in Figure 6a was used, with the indicated blockers added during preincubation. The point in time at which Asc was added is shown by an arrow, and the dash line represents the normalization to baseline (control set to 0.1). (**a**) Preincubation with 20 µM AMG did not significantly change the response compared with the corresponding control measurement (*n* = 18). (**b**) Preincubation with 20 µM AMTB reduced the maximal f340/f380 response at *t* = 600 s (*n* = 37). (**c**) Preincubation with 10 µM GSK219 results in a partial inhibition of f340/f380 signal compared to control measurements (*n* = 14). (**d**) Preincubation with 10 µM AM251 produces an irreversible increase, with lower f340/f380 values than in control measurements (*n* = 38). (**e**) The diagram presents a statistical analysis of the experiments shown in panels (**a**–**d**), compared with the response to 1 mM Asc following preincubation with 10 µM WIN (Figure 6a). Mean values ± SEM are shown at 150 s, 400 s, and 600 s. The numbers in brackets above the columns indicate the number of single cells measured (*n*). Asterisks (*) indicate statistical significance in paired data (*** *p* < 0.001), whereas hashtags (#) indicate statistical significance in unpaired data (### *p* < 0.001). Statistical significance was defined as *p* < 0.05.

Measurements performed after treatment with 1 mM Asc and preincubation with 10 µM WIN in addition to preincubation with specific TRP channel blockers, specifically 20 µM AMG, 20 µM AMTB, 10 µM GSK219, 10 µM AM251 exhibit a response pattern of [Ca^2+^]_i_ increase similar to that of Figure 6a. Meanwhile, overall suppressed increases in [Ca^2+^]_i_ could be observed. Preincubation with 20 µM AMG results in a maximum of f340/f380 at 0.2390 ± 0.0035 (*t* = 600 s after Asc addition; *n* = 18; Figure 7a), compared to 0.2616 ± 0.0072 (*t* = 600 s; *n* = 39; *p* > 0.05; Figure 6a). Experiments with 20 µM AMTB preincubation demonstrate lower f340/f380 values of 0.1537 ± 0.0030 (*t* = 400 s after 1 mM Asc addition; *n* = 37; Figure 7b) and 0.2059 ± 0.0030 (*t* = 600 s; *n* = 37; Figure 7b), compared to control measurements without blocker, which yield values of 0.1884 ± 0.0074 (*t* = 400 s; *n* = 39; *p* < 0.0001; Figure 6a) and 0.2616 ± 0.0072 (*t* = 600 s; *n* = 39; *p* < 0.001; Figure 6a). Similarly, preincubation with 10 µM GSK219 leads to a lower f340/f380 of 0.1022 ± 0.0063 (*t* = 400 s; *n* = 14; Figure 7c) and 0.1603 ± 0.0063 (*t* = 600 s; *n* = 14; Figure 7c) compared to control values shown in Figure 6a). Notably, GSK219 shows the strongest inhibition of Asc- and WIN-induced [Ca^2+^]_i_ signal. Interestingly, preincubation with 10 µM AM251 results in a distinct curve pattern, with the f340/f380 increasing proportionally without approaching a plateau. Nevertheless, the values remain lower than those observed in the control experiment without blocker, at 0.1360 ± 0.0022 (*t* = 400 s following 1 mM Asc addition; *n* = 38; Figure 7d) and 0.1918 ± 0.0021 (*t* = 600 s; *n* = 38; Figure 7d), compared to 0.1884 ± 0.0073 (*t* = 400 s; *n* = 39; *p* < 0.001; Figure 6a) and 0.2616 ± 0.0072 (*t* = 600 s; *n* = 39; *p* < 0.001; Figure 6a).

## 3. Discussion

### 3.1. Role of Asc on [Ca^2+^]_i_

Treatment with 1 mM Asc leads to an immediate increase of [Ca^2+^]_i_ in HCEC-12 cells (Figure 1a). Specific TRPs subtypes, including TRPV1 and TRPV4, are partially involved for the Asc-induced increase in [Ca^2+^]_i_ in HCEC-12 in the short term, as indicated by experiments using selective blockers (Figure 1b–d). While individual blockers were able to partially reduce the increase in [Ca^2+^]_i_, these findings are consistent with previous experiments. For example, in the HEK293T cell line, 2 mM Asc induced TRPV1-mediated transmembrane currents in patch-clamp measurements [50]. In retinoblastoma cells, 1 mM Asc induced Ca^2+^ transients through activation of TRPs. In these cells, Asc decreased viability and induced apoptosis, possibly due to pathological TRPs expression and disrupted Ca^2+^ homeostasis [22]. Altered apoptotic and survival signaling in cancer cells may explain why Ca^2+^ changes have opposite effects in physiological versus tumor cells [23,51,52,53].

Treatment with 1 mM Asc revealed an effect that appears at first glance contradictory to its well-known antioxidative and protective properties in corneal tissue. While acute treatment with 1 mM Asc induced a transient increase in [Ca^2+^]_i_ (Figure 1), washout after 24-h preincubation of Asc resulted in a marked and disproportionate elevation of [Ca^2+^]_i_ (Figure 2). Notably, this increase exceeded the levels observed during continuous Asc presence, indicating a sustained sensitization of Ca^2+^ entry pathways. This finding highlights the critical importance of the time factor in interpreting Asc-mediated Ca^2+^ signaling. Intracellular Ca^2+^ is an essential second messenger regulating proliferation, metabolism, and survival. However, sustained and excessive Ca^2+^ overload is well known to trigger apoptotic pathways [54]. Thus, the biological relevance of Asc-induced Ca^2+^ elevations cannot be evaluated solely based on their occurrence, but rather on their amplitude, duration, and reversibility. In the presence of Asc, Ca^2+^ influx appears transient and may even be followed by compensatory mechanisms that stabilize or reduce [Ca^2+^]_i_. In contrast, removal of Asc after prolonged exposure unmasks an enhanced Ca^2+^ entry, suggesting adaptive or sensitizing processes at the level of TRPs. This effect is further supported by experiments shown in Figure S2, where Asc treatment followed by washout similarly results in an increase in [Ca^2+^]_i_, even under modified extracellular calcium conditions. This indicates that the enhanced Ca^2+^ response is closely linked to Asc exposure and its subsequent removal. Such behavior is consistent with Ca^2+^-dependent desensitization and feedback regulation of TRPs involving calcineurin-mediated dephosphorylation, changes in membrane phosphoinositide availability, or channel internalization during sustained stimulation. Relief of this inhibitory tone after stimulus withdrawal may functionally expose augmented channel activity, thereby contributing to the pronounced Ca^2+^ influx observed after washout [55,56,57]. Alternatively, changes to the level of ion channel transcription are plausible. Alterations in channel expression may be mediated by Ca^2+^-dependent signaling pathways. In neuronal cells, the impact of Ca^2+^ transients on transcription has been described, involving cAMP response element-binding protein (CREB), MAPK, and Ca^2+^/calmodulin-dependent protein kinase I, II, and IV (CaMK I, II, and IV) [58,59]. ERK1/2, activated downstream of CaMK I signaling, has been shown to influence chromatin remodeling in neuronal cells [60,61,62]. In corneal epithelial cells, Ca^2+^-dependent signaling downstream of TRP channel activation has been linked to MAPK pathway activation [37]. However, in contrast to neuronal systems, comparable direct mechanistic evidence linking Ca^2+^ transients to CREB- or CaMK-dependent transcriptional regulation in corneal endothelial cells remains scarce.

### 3.2. Mechanism of Asc Induced TRP Channel Activation and [Ca^2+^]_i_ Increases

Asc-induced increases in [Ca^2+^]_i_ are dependent on extracellular Ca^2+^, as no elevation in [Ca^2+^]_i_ was observed under Ca^2+^-free conditions. This indicates that transmembrane Ca^2+^ influx is the primary contributor to the observed effect (Figure 3; Figure S1). The precise mechanism underlying Asc-induced TRP channel activation and subsequent [Ca^2+^]_i_ elevation in HCEC-12 remains unclear (Figure 1a). Since single selective TRP channel blockers were unable to fully eliminate the [Ca^2+^]_i_ increase (Figure 1b–d), it is possible that additional Ca^2+^ channels and TRPs are involved. In sensory neurons, GPCRs have been shown to sensitize TRPs, providing evidence for a functional interaction between GPCRs and TRPs [29]. In HCECs, Asc-dependent effects on cell proliferation have been reported to depend on GLUT1 activity, which is known to function as a transporter of dehydroascorbic acid [12,19,63]. SVCT1 and SVCT2 are also known to transport Asc (reduced ascorbate) into mammalian cells [64]. Therefore, it would be relevant to investigate whether the effects of Asc on [Ca^2+^]_i_ are associated with its intracellular transport, and whether GLUT1 or other transporters, such as SVCT1/2, play a significant role in this process. At physiological pH (7.4), Asc is predominantly in its reduced form [65,66]. Although ascorbate is generally regarded as an antioxidant in vivo, its behavior in cell culture systems can differ substantially compared to in vitro. In the presence of dissolved oxygen and trace transition metals, extracellular ascorbate can undergo autoxidation. This process generates superoxide and subsequently H_2_O_2_, which can accumulate in the culture medium. Such medium-dependent H_2_O_2_ formation has been identified as a potential confounder in in vitro studies using millimolar ascorbate concentrations [67,68]. H_2_O_2_ is a known activator of TRPA1 in guinea pig bladder, TRPV1 in HEK293A and bovine aortic endothelial cells, TRPM2 in HEK cells, and voltage-gated Ca^2+^-channels in HCECs [24,69,70,71]. Consistent with this, exogenous H_2_O_2_ has been shown to induce dose-dependent increases in [Ca^2+^]_i_ concentration in HCECs, thereby impairing endothelial viability. Therefore, it cannot be excluded that the observed [Ca^2+^]_i_ increase following Asc treatment is, to a limited extent, influenced by H_2_O_2_ formation under our experimental conditions. However, the distinct temporal dynamics and the modulation of the response by specific TRP channel blockers indicate that the observed effects are predominantly mediated by regulated, channel-dependent Ca^2+^ entry pathways rather than nonspecific oxidative mechanisms. Notably, epidermal growth factor (EGF) significantly attenuated H_2_O_2_-induced elevations in [Ca^2+^]_i_ in HCECs, indicating a protective effect on Ca^2+^ homeostasis [71].

Patch-clamp recordings of our study revealed that Asc induced both inward and outward membrane currents, corroborating the Ca^2+^ imaging findings. These results indicate the involvement of non-selective cation channel activation at the cell membrane. Moreover, a trend toward partial reversal of the Asc effect was observed following TRPV1 blockade with AMG, indicating TRPV1 channel involvement (Figure 4). The observed variability in responses after blocker treatment may be attributed to leak currents (see Section 3.6)

### 3.3. CB-Mediated Modulation of [Ca^2+^]_i_

Activation of CB by WIN is associated with an increase in [Ca^2+^]_i_, which is partially inhibited by the CB1 antagonist AM251, suggesting a predominant involvement of CB1 receptors (Figure 5). However, as WIN is not fully selective for CB1 receptors and can also activate CB2, a contribution of CB2-mediated signaling cannot be excluded. The influence of WIN on HCECs has previously been described, demonstrating an increase in [Ca^2+^]_i_ that is consistent with the findings of the present study [38]. However, blockade of TRPV1 with capsazepine (CPZ) was previously reported to enhance the 10 µM WIN-induced increase in [Ca^2+^]_i_ [38], whereas in our experiments TRPV1 blockade with AMG reduced the WIN-induced [Ca^2+^]_i_ response. One possible explanation for this discrepancy is the different pharmacological profile of the two inhibitors. In contrast to AMG, CPZ is not fully selective for TRPV1 and has also been reported to antagonize TRPM8 at concentrations around 10 µM [72]. Therefore, the previously observed effect of CPZ may not reflect isolated TRPV1 blockade but may additionally involve TRPM8-dependent effects or functional interactions between TRPV1 and TRPM8. In human corneal epithelial cells, a similar effect of WIN on [Ca^2+^]_i_ could be observed, as it induced [Ca^2+^]_i_ transients that were blocked by AM251. These transients induced downstream signaling, leading to an increase in cell proliferation and migration [73]. While corresponding findings in HCECs have mainly been described in the context of the aforementioned HCEC-12 studies, these epithelial data provide supportive evidence for CB1-dependent Ca^2+^ signaling mechanisms across different corneal cell types. In retinoblastoma cells and human uveal melanoma cells, 10 µM WIN has also been reported to induce [Ca^2+^]_i_ transients [49,74]. Interestingly, CB1 activation by WIN reduced [Ca^2+^]_i_ increases induced by capsaicin, a TRPV1 agonist, indicating a modulatory interaction between CB1 signaling and TRPV1-mediated Ca^2+^ entry.

### 3.4. WIN Enhances Asc-Induced Ca^2+^ Responses Involving TRPM8 and TRPV4

We also examined the interaction between Asc and WIN regarding their effects on [Ca^2+^]_i_. Interestingly, when both agents were added concurrently, a significant increase in [Ca^2+^]_i_ was observed, exceeding the effect of either agent alone, suggesting an additive interaction (Figure 6). Preincubation with WIN followed by Asc treatment produced a pronounced increase in [Ca^2+^]_i_ levels, whereas simultaneous treatment with Asc and WIN induced an even stronger biphasic response pattern. One possible explanation for these differential outcomes is that preincubation with WIN allowed TRPs to adapt to WIN-induced sensitization, thereby eliciting a more moderate Ca^2+^ response that could still be compensated by the cell, as reflected by the plateau phase observed in the measurements (Figure 6a). In contrast, co-treatment of Asc and WIN may have prevented such adaptive mechanisms, leading to a more pronounced increase in [Ca^2+^]_i_. This excessive Ca^2+^ load may have exceeded the cellular compensatory capacity, resulting in additional net Ca^2+^ influx manifested as a biphasic response pattern (Figure 6b). The inhibitory effects of AMTB, GSK219, and AM251 suggest that TRPM8, TRPV4, and CB signaling are involved in this enhanced response, with CB1 receptors likely contributing most prominently (Figure 7). However, as WIN 55,212-2 is not fully selective for CB1 receptors and can also activate CB2, the contribution of CB2-mediated signaling cannot be excluded. While TRPV1 was shown to contribute to Ca^2+^ signaling under other experimental conditions, its contribution in this specific setting appears to be less pronounced. This may reflect a context-dependent shift in channel involvement, where multiple TRPs contribute in parallel and the relative impact of TRPV1 is masked by dominant TRPM8- and TRPV4-mediated effects. CB1 and TRPV1 have been shown to be co-expressed in mouse corneal tissue and exhibit functional interactions, which can be inhibited by pertussis toxin, a specific GPCRs blocker [37]. WIN has been reported to modulate TRPV1 activity, resulting in reduced cell currents and altered downstream effects in corneal epithelial cells, ultimately leading to a suppressed inflammatory response and enhanced wound healing [36,37]. In neuronal cells, CB1-mediated neuroprotective effects have been associated with the phosphoinositide 3-kinase/protein kinase B/glycogen synthase kinase 3 signaling pathway, and studies have shown that Asc also interacts with this pathway [75]. Asc and CB1 agonists such as WIN have both been shown to promote corneal wound healing; moreover, evidence suggests they may share common signaling mechanisms [12,36,75].

### 3.5. Clinical Relevance

Endothelial dysfunction can lead to corneal opacity and, subsequently, loss of vision [76]. The most common cause of this dysfunction is Fuchs endothelial dystrophy (FECD), followed by postoperative endothelial damage [11]. FECD is a hereditary disease, predominantly with an autosomal-dominant inheritance pattern [77]. The disease is characterized by the pathological aggregations known as guttae. They contribute to progressive corneal endothelial failure, leading to corneal swelling and irreversible vision loss [78,79]. Cellular pathways activated or altered by oxidative stress, such as the TGF-β pathway, are crucial to disease development [80].

Currently, the only available treatment for corneal endothelial dysfunction is keratoplasty; however, the demand for donor tissues exceeds the available supply [11,81]. There is a clear need for alternative therapies, such as pharmaceutical approaches, that could slow disease progression and endothelial cell loss. Additionally, the development of improved storage media for corneal grafts, aimed at enhancing endothelial cell vitality, is critical for better graft survival and patient outcomes. Interestingly, Asc is a component of Optisol-GS, a widely used corneal storage medium in the United States, where it serves as an antioxidant to support endothelial cell viability during preservation [82]. Regarding calcium homeostasis, Asc could potentially have a protective effect, as [Ca^2+^]_i_ tends to be suppressed during (prolonged) treatment of HCECs with Asc, thereby preventing (toxic) calcium overload [54], as suggested in this in vitro study (Figure 2). Notably, endothelial cell density at six months postoperatively has been shown to significantly correlate with ten-year graft survival [11], raising the question of whether postoperative cell density could be influenced through pharmaceutical intervention. Electrophysiological assessments considering TRPs could provide a possible highly sensitive method for evaluation of corneal endothelial cell viability [83]. A recent study demonstrated the effect of 1 mM Asc on the proliferation of immortalized HCECs of the clonal cell line B4G12 in vitro. In addition, the amelioration of Benzalkonium chloride induced corneal endothelial dysfunction in both in vivo and ex vivo experiments following topical treatment of 284 mM Asc [12]. A previous study also demonstrated that Asc reduces oxidative stress-induced apoptosis, leading to a decrease in P62 protein expression and, consequently, inhibition of autolysosome degradation in the B4G12 cell line [17]. In small clinical reports, perioperative 50 mg/mL topical ascorbic acid was used without apparent ocular complications, but robust clinical dose–toxicity studies remain scarce [84]. Activation of CB1 and CB2 in conjunctival epithelial cells has been shown to suppress cAMP levels and increase proliferation rates [85]. In addition, CB signaling may be clinically relevant in corneal pathology beyond the mechanisms examined here. Topical activation of CB has been reported to reduce corneal pain and inflammation and, more recently, CB2 agonism was shown to promote wound closure and modulate inflammation after alkali burn in vivo. These observations support the view that cannabinoid-dependent signaling pathways may represent promising therapeutic targets in corneal disease, although their specific role in corneal endothelial cells remains to be further clarified [86].

### 3.6. Limitations

The limitations of this study can be categorized into biological and technical aspects. Biological limitations include the fact that all experiments were conducted in vitro under standardized conditions using the immortalized HCEC-12 cell line. While this model allows for controlled analyses, it does not fully replicate the physiological environment, including interactions with the aqueous humor or the corneal stroma. Nonetheless, HCEC-12 is a well-established and widely accepted cell line for studies of HCECs as shown, for example, regarding TRPM8 detection using human corneal endothelium (HCE) primary cultivated cells [34]. Additionally, measurements were not performed under physiological conditions. Experiments were conducted at room temperature rather than at 37 °C to avoid unwanted activation of thermosensitive TRPs. Furthermore, CO_2_ levels during measurements reflected atmospheric rather than physiological concentrations. The use of healthy, immortalized cells also limits the extrapolation of findings—particularly regarding Ca^2+^ homeostasis, regulation, and TRPs expression—to cells under pathological or disease conditions. Technical limitations relate to the methodologies employed. The use of WIN as a pharmacological tool represents a limitation, as it is not fully selective for CB1 receptors and may also activate CB2. Therefore, a clear distinction between CB1- and CB2-mediated effects is not possible under the present experimental conditions. Fura-2 AM calcium imaging and planar patch-clamp techniques were used to investigate [Ca^2+^]_i_ dynamics and whole-cell membrane currents in HCEC-12 cells. Drift correction using TIDA software was applied to compensate for bleaching effects in fluorescence measurements. Preparing HCEC-12 cells for measurement required multiple wash steps and brief exposure outside of solution during transfer to the measurement chamber. This may have introduced cellular stress and contributed to variability in [Ca^2+^]_i_ signals. To mitigate this, care was taken to minimize manipulation and limit the duration of time the cells were not in solution. While patch-clamp recordings provide reliable data, interpretation must be approached with caution. Real-time assessment of seal quality during measurement is not directly possible, posing the risk that leak currents could mimic true transmembrane currents. During experiments, achieving and maintaining high-resistance seals often required interruptions especially after solution changes during the recordings to (e.g., leak current compensation). Therefore, measurements were briefly interrupted to restore the seal’s stability (x-axis brake in Figure 4a). Notably, experiments conducted with AMG frequently led to disruption of the cell seal, which may account for the heterogeneous results observed across individual measurements. Other TRP channel blockers, such as GSK-219, were not included in the patch-clamp experiments due to technical limitations, as stable seals could not be reliably achieved under these conditions. Notably, the mean access resistance in our patch-clamp recordings was in the higher acceptable range. Therefore, a minor impact on the quantitative precision of certain electrophysiological parameters cannot be fully excluded and should be considered when interpreting the results. Despite these limitations, the study has several strengths. The use of well-established techniques and a high number of replicates across most experiments increases the reliability of the observed effects, reducing the likelihood of data distortion. Moreover, all procedures were conducted in a standardized manner, minimizing the influence of potential confounding variables.

## 4. Materials and Methods

### 4.1. Cell Culture

As a cell model for HCE, the SV40-transfected and immortalized HCEC-12 cell line was used, which was kindly provided by Monika Valtink (Faculty of Medicine, Institute of Anatomy, TU Dresden, 01216 Dresden, Germany). Cell culture was performed as previously described [32,33]. Briefly, the cells were grown in sterile-filtered Dulbecco’s Modified Eagle Medium, supplemented with sterile-filtered penicillin/streptomycin antibiotics (100 IU/mL) and 10% fetal bovine serum. Cell culture media and supplements were sourced from Life Technologies Invitrogen (Karlsruhe, Germany) or Biochrom AG (Berlin, Germany). The medium was changed three times per week. The cells were cultured in a humidified environment at 37 °C in an incubator containing 5% CO_2_. Cell passaging was conducted by trypsinization, with trypsin activity being terminated using serum-supplemented growth medium. All additional reagents were purchased from Sigma (Deisenhofen, Germany). For calcium imaging, cells were seeded in 12-well plates with coverslips. To prepare cells for patch-clamping, a high-density single-cell suspension was used.

### 4.2. Fluorescence Calcium Imaging

Cells grown on coverslips were loaded with fura-2 AM (1 µM; TOCRIS Bioscience, Bristol, UK), a ratiometric fluorescent Ca^2+^ indicator used to measure [Ca^2+^]_i_ levels, by incubation in culture medium for 20–40 min at 37 °C [87].

Following dye loading, the coverslips were washed and maintained in a RLS consisting of 150 mM NaCl, 6 mM CsCl, 1.5 mM CaCl_2_, 1 mM MgCl_2_, 10 mM glucose, and 10 mM HEPES (pH 7.4, 317 mOsmol/L). In experiments where pharmacological agents were dissolved in dimethyl sulfoxide (DMSO), the final solvent concentration did not exceed 0.1%. At this concentration, DMSO is generally considered not to interfere with fluorescence-based Ca^2+^ measurements [88,89].

Fluorescence measurements were performed using an inverted fluorescence microscope (Olympus BW50WI, Olympus Europa Holding GmbH, Hamburg, Germany) equipped with a software-controlled high-intensity LED light source (Omikron V1.0, Rodgau-Dudenhofen, Germany) and a monochrome digital camera (Olympus XM10, Olympus, Hamburg, Germany). Image acquisition and hardware control were managed via a U-RTC real-time controller (Olympus Europa Holding GmbH, Hamburg, Germany) connected to a Windows-based computer running cellSens Dimension life imaging software (version 1.16, Olympus Europa Holding GmbH, Hamburg, Germany) [34].

For Ca^2+^ measurements, fluorescence signals were recorded at excitation wavelengths of 340 nm and 380 nm, with emission collected at 510 nm. Regions of interest (ROIs) corresponding to individual cells were defined manually, and f340/f380, reflecting [Ca^2+^]_i_ concentration, was calculated for each ROI using the cellSens software (version 1.16). The fluorescence ratios were normalized to baseline conditions (control set to 0.1) to enable comparison across experiments. To compensate for signal drift, particularly due to photobleaching during prolonged recordings, data were corrected using TIDA software (version 5.25, HEKA Electronic, Lamprecht, Germany) [47].

### 4.3. Planar Patch-Clamping

Electrophysiological measurements were performed using a planar patch-clamp setup, specifically a high-throughput patch-clamp system (Nanion Technologies GmbH; Nanion^®^, Munich, Germany) as described by Brüggemann et al. [90]. Initially, an internal solution containing 50 mM CsCl, 10 mM NaCl, 60 mM CsF, 20 mM EGTA, and 10 mM HEPES, pH 7.2 (adjusted with KOH, osmolarity: 288 mOsmol), was added to inner part of a measurement chip with a resistance of 2.5–3 MΩ. This chip was then mounted onto the setup, establishing a sealed environment. Subsequently, an external solution, consisting of 140 mM NaCl, 4 mM KCl, 1 mM MgCl_2_, 2 mM CaCl_2_, 5 mM D-glucose monohydrate, and 10 mM HEPES, pH 7.4 (adjusted with NaOH, osmolarity: 298 mOsmol), was added on top of the chip as the standard external solution. Both solutions were provided by Nanion Technologies GmbH; Nanion^®^, Munich, Germany). The prepared single-cell suspension was then pipetted on top of the external solution. The system automatically applied suction to the cells by a pump, which was controlled by the PatchMaster software Version 2.73 (HEKA Electronic, Lamprecht/Pfalz, Germany), allowing for the establishment and maintenance of a cell seal. Suction pulses were employed to disrupt the cell membrane, opening it to the aperture and enabling whole-cell configuration measurements. To enhance the seal, a seal enhancer solution (20 μL), containing additional Ca^2+^, was introduced. Membrane currents were recorded using an EPC 10 amplifier with PatchMaster software for Windows Version 2.73 (HEKA Electronic, Lamprecht/Pfalz, Germany). Membrane capacitance (12.53 ± 1.06 pF; *n* = 19) and access resistance (28.32 ± 3.40 MΩ; *n* = 19) were calculated using the PatchMaster software. Electrophysiological characterization of the cells was performed by applying voltage pulse protocols and perfusing appropriate compounds to study specific ion channel behavior, as described by Brüggemann et al. (2006) [90]. Membrane currents were measured with cells held at 0 mV, and current–voltage relationships were obtained from voltage ramps ranging from –60 to +130 mV (duration 500 ms) applied at a frequency of 1 Hz. Data were analyzed and visualized using SigmaPlot Software version 12.5 for Windows (Systat Software, Inc., Point Richmond, CA, USA), including the electrophysiology module, and bar charts were generated using GraphPad Prism software version 5.00 for Windows (La Jolla, CA, USA).

### 4.4. Data Analyses and Statistics

All data are presented as mean ± Standard Error of the Mean (SEM). Normality of data distribution was assessed using the Shapiro–Wilk test or, where appropriate, the Kolmogorov–Smirnov test. Depending on the distribution and experimental design, different statistical analyses were applied. For comparisons between two groups, parametric paired data were analyzed using a paired Student’s *t*-test, whereas parametric unpaired data were evaluated using an unpaired Student’s *t*-test. Non-parametric paired data were analyzed using the Wilcoxon signed-rank test, and non-parametric unpaired data were assessed using the Mann–Whitney U test. For comparisons involving more than two groups, normally distributed data were analyzed by one-way analysis of variance (one-way ANOVA). When data did not meet the assumptions of normality, the Kruskal–Wallis test was applied. In cases where overall significance was detected, appropriate multiple-comparison post hoc tests were performed (e.g., Dunn’s post hoc test for non-parametric data). Statistical significance was defined as *p* < 0.05 (*). All analyses were performed using GraphPad Prism software (version 5.0; San Diego, CA, USA). Regarding the bar charts, mean values ± SEM in both directions are shown at certain points in time as indicated in the diagrams. Statistical significance was indicated using asterisks * for *p* < 0.05, ** for *p* < 0.01, and *** for *p* < 0.001. The numbers in brackets above the columns indicate the number of single cells measured (n). Asterisks (*) indicate statistical significance in paired data, while hashtags (#) indicate statistical significance in unpaired data. Statistical significance was defined as *p* < 0.05.

## 5. Conclusions

**Figure 8:**
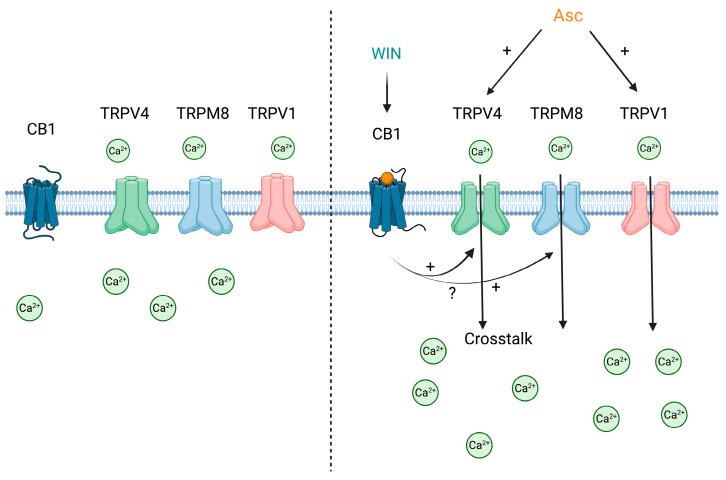
Schematic summary of Asc- and WIN-induced Ca^2+^ signaling in HCEC-12 cells. The left panel illustrates TRPV4 (green), TRPM8 (blue), and TRPV1 (red) channels in their inactive states at the cell membrane. In this scenario, the cell is in RLS, the channels remain closed, and no Ca^2+^ influx is observed. The CB1 receptor (dark blue) is also shown in its inactive state, as no ligand is bound. The right panel depicts the same channels following the treatment with Asc in RLS. TRPV4 and TRPV1 are activated, resulting in a significant Ca^2+^ influx. Simultaneous treatment with WIN leads to CB activation, which induces interactions with TRPV4 and TRPM8. This CB–TRPs interaction enhances TRP channel activation, resulting in additive effects compared to the treatment with WIN or Asc alone. The precise mechanism underlying the crosstalk between CB and TRPs remains unknown. Green circles indicate Ca^2+^ ions, straight black arrows indicate Ca^2+^ influx, black arrows with plus signs indicate activation or enhancement, curved arrows indicate potential crosstalk, and the question mark indicates mechanistic uncertainty.

Using fluorescence Ca^2+^ imaging and the planar patch-clamp recordings, we first demonstrate that 1 mM Asc immediately increases [Ca^2+^]_i_ in HCEC-12 cells, as indicated by the effects of specific TRPV1 and TRPV4 channel blockers. Notably, our results suggest sustained sensitization of TRP-mediated signaling, as washout following 24-h Asc incubation resulted in a marked increase in [Ca^2+^]_i_. This observation suggests that prolonged Asc exposure may alter [Ca^2+^]_i_ regulation, potentially leading to a relative suppression of Ca^2+^ levels during treatment. Furthermore, WIN-induced Ca^2+^ transients were modulated by TRP channel blockers, suggesting a functional interaction between CB signaling and TRPs, including TRPV1, TRPV4, and TRPM8. While inhibition by the CB1 antagonist AM251 supports a predominant involvement of CB1 receptors, a contribution of CB2-mediated signaling cannot be excluded. Importantly, combined treatment with Asc and WIN resulted in enhanced [Ca^2+^]_i_ elevations, indicating an additive effect. This suggests functional interaction between TRPs and CB signaling pathways (Figure 8). These effects were attenuated by TRPM8 and TRPV4 blockers as well as by CB1 receptor inhibition. Future studies should combine selective pharmacological approaches with genetic and molecular analyses to further characterize the underlying signaling pathways and channel interactions. In summary, our findings provide a foundation for future exploration of Asc- and WIN-induced Ca^2+^-dependent signaling pathways and their potential relevance for corneal endothelial function and pathology.

## Data Availability

The data presented in this study are available on request from the corresponding author.

## Supplementary Materials

The following supporting information can be downloaded at: https://www.mdpi.com/article/10.3390/ijms27093902/s1.

## Abbreviations

The following abbreviations are used in this manuscript:

HCE	Human corneal endothelium
[Ca^2+^]_i_	Intracellular Ca^2+^
TRPs	Transient receptor potential channels
CB1	Cannabinoid receptor 1
CB	Cannabinoid receptor
Asc	L-Ascorbic acid
WIN	WIN 55,212-2
AMG	AMG-9810
AMTB	N-(3-aminopropyl)-2-[(3-methylphenyl) methoxy] -N-(2-thienylmethyl) benzamide hydrochloride
GSK219	GSK2193874
HCECs	Human corneal endothelial cells
GLUT1	Glucose transporter 1
TRPA	Transient receptor potential channel ankyrin subfamily
TRPC	Transient receptor potential channel canonical subfamily
TRPM	Transient receptor potential channel melastatin subfamily
TRPML	Transient receptor potential channel mucolipin subfamily
TRPN	Transient receptor potential channel NO-mechano-potential subfamily
TRPP	Transient receptor potential channel polycystin subfamily
TRPV	Transient receptor potential channel vanilloid subfamily
GPCRs	G protein-coupled receptors
MAPK	Mitogen-activated protein kinases
ERK 1/2	Extracellular Signal-regulated Kinase 1/2
CB2	Cannabinoid receptor 2
f340/f380	Fura-2/AM f340/f380 ratio
SEM	Standard error
CREB	cAMP response element-binding protein
CaMK I, II, IV	Ca^2+^/calmodulin-dependent protein kinase I, II, IV
CPZ	Capsazepine
FECD	Fuchs endothelial dystrophy
RLS	Ringer-Like-Solution
DMSO	Dimethyl sulfoxide
