# Supplementary material for: TRP-Dependent Calcium Regulation in HCEC-12 Cells: Involvement of Ascorbic Acid and Cannabinoid Receptor Signaling
Source: Int J Mol Sci. 2026 Apr 28;27(9):3902. doi: 10.3390/ijms27093902 (PMC13163926; doi:10.3390/ijms27093902)
Supplement: Supplementary file 1 [file ijms-27-03902-s001.zip › ijms-4226295-supplementary.pdf]

Supplementary Materials:

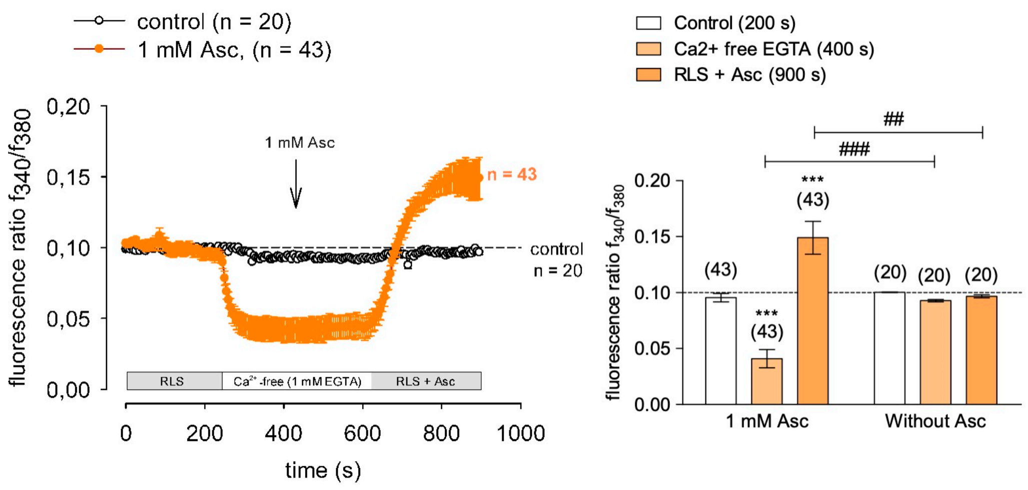

(a) **(b)**

Figure S1: Effect of Asc in extracellular calcium-free conditions. Time-dependent changes are shown as relative intracellular Ca^{2+} levels in Fura-2–loaded cells. The number (*n*) indicates the number of cells examined in this set of experiments. The bars below denote the timing of solution changes and the arrow shows the point in time when Asc was added. The fluorescence ratios were normalized to baseline conditions (control set to 0.1, dashed line). **(a)** Treatment with calcium-free EGTA (1 mM) solution at 240 s induced a decrease in $[Ca^{2+}]_i$ in treated cells (*n* = 43; orange filled circles), while the control measurement showed stable intracellular Ca^{2+} levels (*n* = 20; black open circles). Subsequent addition of 1 mM Asc (t = 420 s) in calcium-free EGTA solution did not alter $[Ca^{2+}]_i$. Re-addition of Asc together with RLS at 600 s produced a pronounced increase in $[Ca^{2+}]_i$ compared to the control condition without Asc. **(b)** Statistical analysis of the Asc-induced Ca^{2+} responses. Columns represent mean values ± SEM of the fluorescence ratio (f340/f380) at 200 s (control), 400 s (calcium-free EGTA), and 900 s (RLS + 1 mM Asc). Numbers in brackets indicate the number of single cells measured (*n*). The dashed line represents the baseline reference level. Asterisks (*)

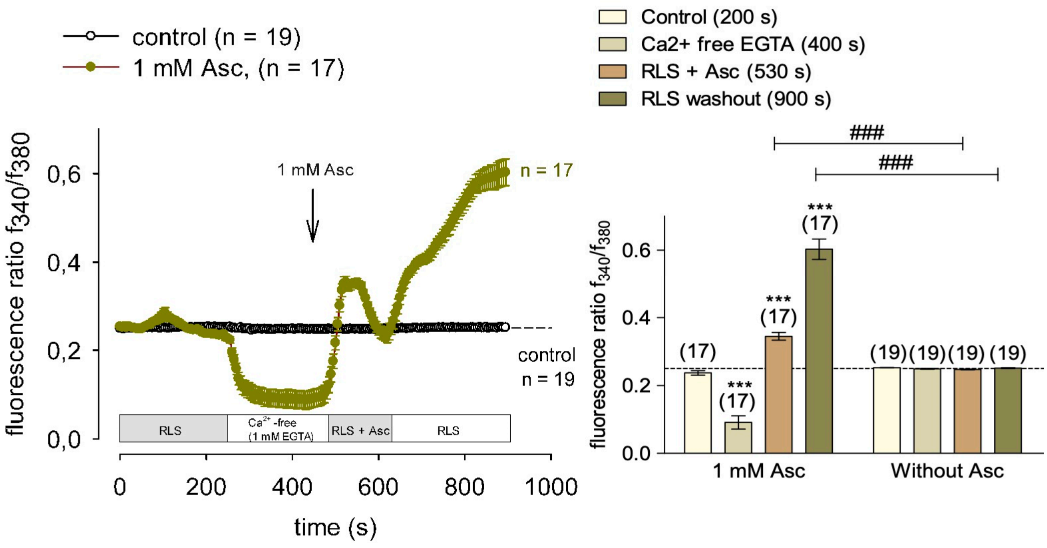

(a) **(b)**

indicate statistically significant differences in paired comparisons (*$p < 0.05$), while hashtags (#) denote significance in unpaired comparisons (#$p < 0.05$).

Figure S2: Effect of Asc following extracellular calcium removal. Time-dependent changes are shown as relative intracellular Ca^{2+} levels in Fura-2–loaded cells. The number (*n*) indicates the number of cells examined in this set of experiments. The bars below denote the timing of solution changes and the arrow shows the point in time when Asc was added. The fluorescence ratios were normalized to baseline conditions (control set to 0.25, dashed line). **(a)** Cells were initially superfused with RLS (0–240 s) to establish a stable baseline $[Ca^{2+}]_i$. Treatment with calcium-free EGTA (1 mM) solution at 240 s induced a clear decrease in $[Ca^{2+}]_i$ in treated cells ($n = 17$; filled green circles), whereas the control measurement ($n = 19$; open circles) showed no obvious change or only a slight reduction in $[Ca^{2+}]_i$. Subsequent addition of 1 mM Asc together with RLS at 420 s produced a transient increase in $[Ca^{2+}]_i$. Washout of Asc by perfusion with RLS alone at 600 s resulted in a pronounced rise in $[Ca^{2+}]_i$ compared to the control condition without Asc. **(b)** Statistical analysis of the Asc-dependent Ca^{2+} responses. Columns represent mean values ± SEM of the fluorescence ratio (f340/f380) measured at baseline (200 s), during calcium-free EGTA (400 s), upon RLS and Asc exposure (530 s), and after RLS washout (900 s). Numbers in brackets indicate the number of single cells measured. The dashed line represents the baseline reference level. Asterisks (*) indicate statistically significant differences in paired comparisons (*$p < 0.05$), while hashtags (#) denote significance in unpaired comparisons (#$p < 0.05$).
